# Prognostic factors among single primary gliosarcoma cases: A study using Surveillance, Epidemiology, and End Results data from 1973–2013

**DOI:** 10.1002/cam4.2503

**Published:** 2019-08-28

**Authors:** Bin Chen, Bin Liu, Chao Wu, Zhenyu Wang

**Affiliations:** ^1^ Department of Neurosurgery Peking University Third Hospital Beijing People's Republic of China

**Keywords:** demographics, SEER, single primary gliosarcoma, survival rate

## Abstract

**Background:**

Prognostic factors for single primary gliosarcoma (PGS) remain unknown.

**Objective:**

The purpose of our study was to examine patient, tumor, and treatment characteristics as potential predictors of survival using Surveillance, Epidemiology, and End Results (SEER) program data (1973‐2013).

**Methods:**

The patients of single PGS were selected based on the exclusion criteria from SEER. Kaplan‐Meier survival analysis, log‐rank test and Cox proportional hazards models were used to analyze all the data.

**Results:**

Single PGS has an apparent popularity for the temporal lobe (35.2%, hazard ratio [HR] = 0.440, 95%CI = 0.251‐0.770) and frontal lobe (20.9%, HR = 0.408, 95%CI = 0.231‐0.720) which could achieve a better survival rate than cerebrum (*P* = .034). The mean age at diagnosis was 60.07 ± 14.161. The overall 6‐month, 1‐year, 2‐year, and 5‐year survival was 55.40%, 29.58%, 10.01%, and 2.73%. Age at diagnosis was proved to be a significant predictor of overall survival (OS) (*P* < .001). There is no significant difference in race, marital status, or grade. Patients' tumor size which is located in 41‐60 mm (*P* = .047, HR = 1.468, 95%CI = 1.004‐2.147) and >60 mm (*P*= .003, HR = 1.899, 95%CI = 1.244‐2.901) showed a higher risk of death. Surgery played a critical role in OS (*P* < .001). Radiation after surgery was another predictor of OS of PGS (*P* < .001). Among all the radiation methods, combination of beam with implants or isotopes (*P* = .000, HR = 0.491, 95%CI = 0.412‐0.585) or radiation NOS (*P* = .027, HR = 0.362, 95%CI = 0.148‐0.889) were more beneficial to patients.

**Conclusion:**

This study indicated that single PGS has a poor prognosis. Prognosis of single PGS would become poorer along with patients' age and tumor size (>40 mm). Surgery intervention and radiation therapy were beneficial factors.

## Graphical abstract

This is the first large sample study of single primary gliosarcoma to examine patient, tumor, and treatment characteristics as potential predictors of survival using Surveillance, Epidemiology, and End Results (SEER) program data (1973‐2013). This study indicated that outcome of single PGS would become poorer along with patients' age getting older. Surgery intervention and radiation therapy were beneficial factors.

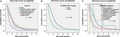

## 1 INTRODUCTION

Primary gliosarcoma (PGS) is a rare malignant tumor of the central nervous system composed of both malignant glial and sarcomatous elements. PGS was first reported by Strobe in 1895 and gradually understood until 1955 by Feigen and Gross's description.^1^, ^2^ Primary gliosarcoma was considered as a fusion of two independent tumors: gliomatous and sarcomatous (mesenchymal) components.^3^ There are several theories to explain the origin of sarcomatous. One theory believed that sarcoma component derived from endothelial cells,^4^ another theory believed that sarcoma components arose from pluripotent mesenchymal cells.^5^ Both the glial component and sarcoma component are malignant and motivate PGS to possess potential to metastasis to distal part of our body. The most common metastasis sites are the lung and liver and some other sites including the spleen, cervical lymph node.^6^

The incidence of PGS is between 1% and 8% in all gliomas representing that it is an exceptionally rare neoplasm.^7^ Primary gliosarcoma and glioblastoma (GBM) share some similarities in terms of symptoms and treatment methods.^8^ However, recent research proposed that there were still distinctions between PGS and GBM. For example, Meis reported that survival of GS (8.3 months) is worse than that of GBM (9.6 months).^9^ Kevin R. Kozak also found that survival of GS was worse than GBM, which is opposite to Lutterbach.^10^, ^11^ Considering its rarity and poor prognosis, our knowledge about this tumor is limited to small retrospective case series and case reports. It is challenging to identify the prognosis of PGS. As a result, in our research, using the Surveillance, Epidemiology, and End Results (SEER) database enabled us to describe the overall survival (OS) rate and prognostic factors of PGS patients. A cohort of single PGS patients (879) diagnosed between 1973 and 2013 were enrolled into this study and were examined in detail.

## 2 METHODS

### 2.1 Study population

This study was designed as a population‐based longitudinal cohort study. Data were obtained from the SEER database (SEER 18—Registries research data and Hurricane Katina Impacted Louisiana Cases between 1973 and 2013). Histological classification was based on the International Classification of Diseases for Oncology (ICD) code. All patients diagnosed as gliosarcoma (GSM) (ICD‐0‐3 9442/3), GBM, not otherwise specified (NOS) (ICD‐0‐3 9440/3), glioma (ICD‐0‐3 9380‐3), mixed glioma (ICD‐0‐3 9382/3), and giant cell GBM (ICD‐0‐3 9441/3) between January 1973 and December 2013 were identified. Exclusion criteria were as follows: (a) histological classification of nongliosarcoma; (b) survival month is unknown; (c) sequence number that is not “one primary only.”

One of the main outcome was OS, which was defined as the number of months from the date of diagnosis to the date of last follow‐up or death. Cause‐specific survival was also examined. Data collected for each subject included age at diagnosis, gender, race, marital status, grade, tumor size, tumor extension, extent of surgical resection, use of radiation therapy after surgery. Chemotherapy was not included in the SEER database.^12^

The SEER program provides information on cancer statistics in an effort to reduce the cancer burden among the US population. It is one of the most representative large‐scale tumor registration databases in North America, providing systematic evidence support and valuable first‐hand information for clinicians. Surveillance, Epidemiology, and End Results is an authoritative source for cancer statistics in the United States and is open for all the clinicians all over the world, so there is no need to ask for patients' approval.

### 2.2 Statistical analysis

The patient populations were evaluated in terms of tumor and treatment characteristics (age at diagnosis, gender, race, tumor size, tumor extension, type of surgery, and radiation therapy after surgery). The OS was assessed as the end point of this study, with patients investigated either at death or at date of last follow‐up. Overall survival was estimated using the Kaplan‐Meier survival analysis, and the log‐rank test (Mantel‐Cox) was used to examine the significance of differences between survival curves. Proportional hazards ratios were assessed using Cox proportional hazards models to calculate hazard ratios (HRs) and their 95% confidence intervals (CIs).

For most analyses, age at diagnosis was categorized as “≤50,” “50‐60,” “60‐70,” “>70.” Race was divided into three categories “white,” “Black,” and “others.” Marital status was categorized into following six types: “married (including common law),” “divorced,” “single (never married),” “separated,” “widowed,” and “unknown.” Grade was divided into five groups, namely “well differentiated,” “moderately differentiated,” “poor differentiated,” “undifferentiated,” and “unknown.” Tumor location radiation method was recorded as same as it is in SEER date. Tumor size was dichotomized into five groups: “≤20 mm,” “21‐40 mm,” “41‐60 mm,” “＞60 mm,” and “unknown.” Tumor extension was categorized into three groups, namely “localized at one side, not cross the midline,” “invasive, cross the midline and distal metastasis,” and “unknown.” The type of surgery was divided into the following six groups: “none,” “excision of tumor, lesion,” “partial resection NOS,” “gross resection,” “radical resection,” and “surgery NOS” according to SEER site‐specific coding guideline‐Appendix C site‐specific surgery codes mentioned in previous research.^13^

SEER*STAT version 8.3.5 (Surveillance Research Program, NCI, Bethesda, MD, USA) was used to extract case data from the SEER public‐use databases.^14^ All analyses were conducted using the Statistical Package for the Social Sciences (IBM SPSS, v19.0; IBM Corporation). *P* < .05 was considered statistically significant.

## 3 RESULTS

### 3.1 Patient population

The study population consisted of 65 221 patients diagnosed with GBM or glioma. The number of PGS patients confirmed by histological diagnosis is 1055 patients. According to our exclusion criteria, 176 patients were excluded and 879 single PGS cases were remained in our research. Primary site of single PGS is displayed in Table 1. Primary gliosarcoma has an apparent popularity for the temporal lobe (35.2%). Following sites are frontal lobe, overlapping lesion of brain and parietal lobe which accounted for 20.9%, 16.8%, and 13.4%, respectively. The least common occurrence is optic nerve and brain stem.

**Table 1:** Anatomical location of single primary gliosarcoma, 1973‐2013 (n = 879)

	Frequency	Percent
Cerebrum	15	1.7
Frontal lobe	184	20.9
Temporal lobe	309	35.2
Parietal lobe	118	13.4
Occipital lobe	41	4.7
Ventricle, NOS	11	1.3
Cerebellum, NOS	9	1.0
Brain stem	2	0.2
Overlapping lesion of brain	148	16.8
Brain, NOS	41	4.7
Optic nerve	1	0.1
Total	879	100.0

Abbreviation: NOS, not otherwise specified.

Table 2 presents general information including age at diagnosis, gender, tumor size, and tumor extension and other variants are displayed. The mean age at diagnosis of all the patients was 60.07 ± 14.161. The white was predominated (87.8%). Gender distribution was the male 61.5% and the female 38.5%. There were more patients' tumor size landed in “41‐60 mm” group (32.4%) and “21‐40 mm” group (26.6%). Most patients' tumor (77.8%) was localized at one side and did not cross the midline. A large number of patients underwent surgeries including excision of tumor, lesion (27.6%), partial resection (16.5%), gross resection (17.9%), and radical resection (28%). Nearly 70% (69.2%) patients were given radiation after surgery.

**Table 2:** General information of all the single primary gliosarcoma patients

Parameters	Total n = 879
Age at diagnosis, mean ± SD	60.07 ± 14.161
Sex, number (%)	
Male	541 (61.5%)
Female	338 (38.5%)
Race, number (%)	
White	772 (87.8%)
Black	62 (7.1%)
Others	45 (5.1%)
Marital status, number (%)	
Married	323 (65.5%)
Divorced	40 (8.1%)
Single	59 (12.0%)
Separated	6 (1.2%)
Widowed	51 (10.3%)
Unknown	14 (2.8%)
Tumor size, mm, number (%)	
≤20 mm	37 (4.2%)
21‐40 mm	234 (26.6%)
41‐60 mm	285 (32.4%)
>60 mm	94 (10.7%)
Unknown	229 (26.1%)
Tumor extension, number (%)	
Localized at one side, not cross the mid line	684 (77.8%)
Invasive, cross the mid line and distal metastasis	135 (15.4%)
Unknown	60 (6.8%)
Type of surgery, number (%)	
None	49 (5.57%)
Excision of tumor, lesion	243 (27.65%)
Partial resection NOS	145 (16.50%)
Gross resection	157 (17.86%)
Radical resection	246 (27.99%)
Surgery NOS	39 (4.44%)
Radiation after surgery, number (%)	
Yes	608 (69.17%)
No	271 (30.83%)

Abbreviations: NOS, not otherwise specified; SD, standard deviation.

### 3.2 Uni‐ and multivariate analyses

**Figure 1:**
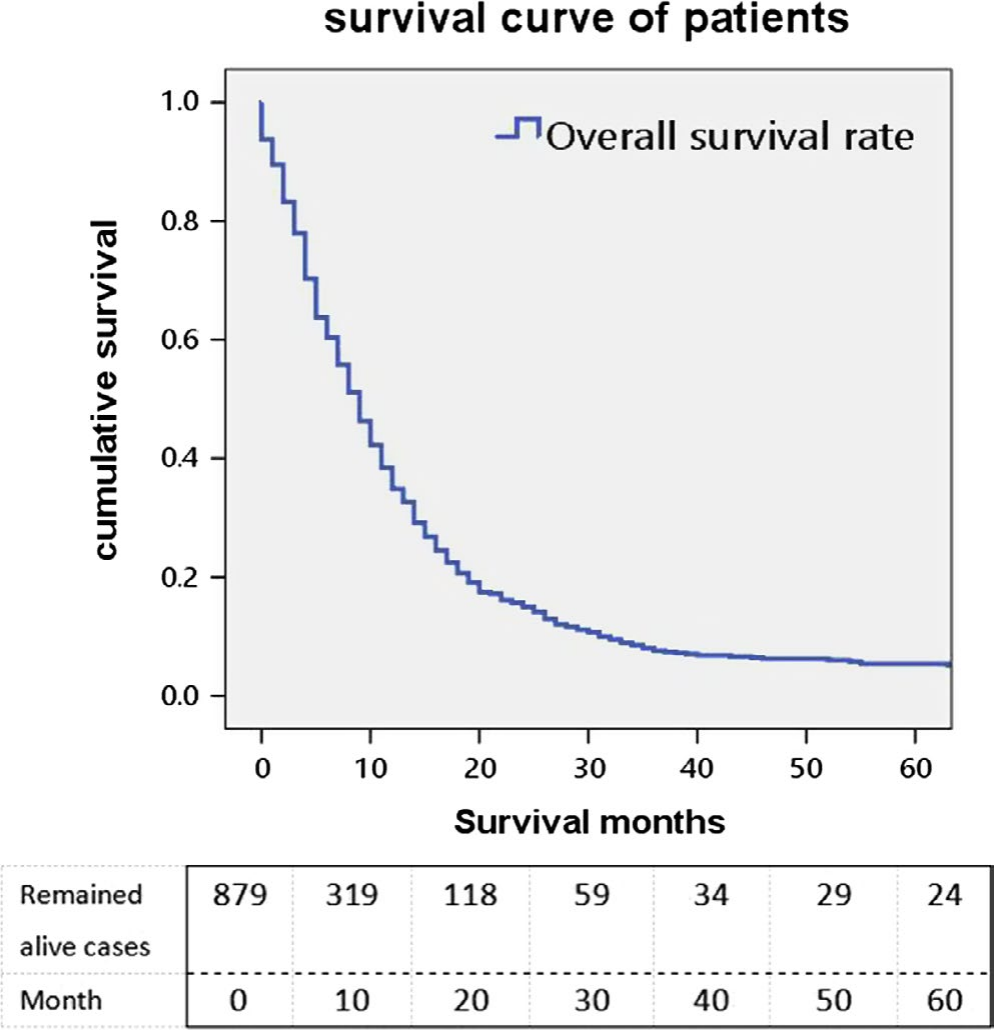
Kaplan‐Meier overall survival curves for entire cohort single primary gliosarcoma patients from the Surveillance, Epidemiology, and End Results database 1973‐2013. Median survival was 9 mo and mean survival month was 12.29 ± 19.078 mo

The evaluation index of this study was tumor‐related death. The 6‐month, 1‐year, 2‐year, and 5‐year survival was 55.40%, 29.58%, 10.01%, and 2.73% (Figure 1). It is apparent that the prognosis of PGS is poor.^15^ As is shown in the univariate Cox proportional analysis model (Table 3), age at diagnosis, tumor size, tumor extension, tumor location, type of surgery, radiation after surgery, and radiation method were significantly associated with survival of single PGS. Age at diagnosis was proved to be a significant predictor of OS when analyzed by univariate method (*P* < .001). Compared to age ≤50 years, 60‐70, and over 70 had an obvious increased risk of death (HR = 1.496, 95%CI = 1.214‐1.843; HR = 2.607, 95%CI = 2.103‐3.232). There is no significant difference in gender, race, marital status, or grade. Tumors located at frontal lobe (HR = 0.408, 95%CI = 0.231‐0.720), temporal lobe (HR = 0.440, 95%CI = 0.251‐0.770), parietal lobe (HR = 0.445, 95%CI = 0.249‐0.794), occipital lobe (HR = 0.440, 95%CI = 0.234‐0.829), cerebellum NOS (HR = 0.411, 95%CI = 0.170‐0.995), and overlapping lesion of brain (HR = 0.521, 95%CI = 0.294‐0.924) showed a relatively better survival rate than cerebrum which had worst prognosis in PGS. The larger tumor size is the higher risks on OS of single PGS. Surgery played a critical role in OS (*P* < .001). Compared to none surgery group, other surgery groups reached statistical significance. Radiation after surgery was another predictor of OS of single PGS (*P* < .001). Among all the radiation methods, combination of beam with implants or isotopes (HR = 0.375, 95%CI = 0.154‐0.911) or beam radiation (HR = 0.476, 95%CI = 0.404‐0.562) were more beneficial to patients.

**Table 3:** Results of univariate analysis using the Cox proportional hazards model

Variances	*P* value	Hazards ratio	95% confidence interval
Age	**<.001**		
≤50		1	
50‐60	.204	1.151	0.926‐1.431
60‐70	**.000**	1.496	1.214‐1.843
>70	**.000**	2.607	2.103‐3.232
Gender	.338		
Male		1	
Female	.338	1.075	0.927‐1.247
Race	.461		
White		1	
Black	.624	0.927	0.685‐1.255
Others	.242	0.822	0.592‐1.141
Marital status	.343		
Married (including common law)		1	
Divorced	.400	0.858	0.600‐1.226
Single (never married)	.101	1.277	0.953‐1.711
Separated	.808	0.885	0.329‐2.376
Widowed	.859	1.029	0.751‐1.410
Unknown	.212	0.681	0.373‐1.245
Grade	.598		
Well differentiated		1	
Moderately differentiated	.979	1.031	0.107‐9.921
Poor differentiated	.560	0.553	0.075‐4.072
Undifferentiated	.471	0.485	0.068
Unknown	.462	0.478	0.067
Tumor size, mm	**.006**		
≤20 mm		1	
21‐40 mm	.769	1.058	0.728‐1.538
41‐60 mm	.547	1.120	0.774‐1.623
>60 mm	.152	1.352	0.895‐2.042
Unknown	**.043**	1.469	1.011‐2.135
Tumor extension	**.010**		
Localized at one side, not cross the mid line		1	
Invasive, cross the mid line and distal metastasis	**.045**	1.229	1.005‐1.504
Unknown	**.013**	1.415	1.077‐1.859
Type of surgery	**.000**		
None		1	
Partial resection	**.000**	0.433	0.316‐0.594
Partial resection NOS	**.000**	0.420	0.299‐0.590
Gross resection	**.000**	0.489	0.353‐0.678
Radical resection	**.000**	0.419	0.307‐0.573
Surgery NOS	**.009**	0.565	0.367‐0.869
Radiation after surgery	**.000**		
Yes		1	
No	**.000**	2.090	1.789‐2.442

Bold indicates statistically significance.

Abbreviation: NOS, not otherwise specified.

Those demographics, clinical characteristics and treatment variables whose *P* value <0.2 were analyzed using multivariate Cox proportional analysis model (Table 4). Age at diagnosis was a critical prognostic factor of single PGS patients. Compared to age ≤50 years, which was used as reference, patients aged 50‐60, 60‐70, and over 70 had an obvious increased risk of death (HR = 1.467, 95%CI = 1.165‐1.847; HR = 1.847, 95%CI = 1.481‐2.303; HR = 3.154, 95%CI = 2.503‐3.975). Radical resection, gross resection, partial resection (compared to no surgery) were associated with higher survival rate (HR = 0.560, 95%CI = 0.375‐0.835; HR = 0.574, 95%CI = 0.381‐0.865; HR = 0.572, 95%CI = 0.387‐0.845). There is no significant difference was observed in terms of gender, race, marital status, grade, tumor extension, radiation after surgery, and location. However, among the radiation methods, beam radiation (HR = 0.0.639, 95%CI = 0.426‐0.959) showed beneficial effects to PGS patients. In the final model, we found that age at diagnosis, type of surgery, tumor size, and radiation method (combination of beam with implants or isotopes, HR = 0.362, 95%CI = 0.148‐0.889; radiation, NOS method or source not specified, HR = 0.491, 95%CI = 0.412‐0.585) were confirmed to be independent influencing factors to the prognosis of single PGS (Table 5).

**Table 4:** Results of multivariate analysis using the Cox proportional hazards model

Variances	*P* value	Hazards ratio	95% confidence interval
Age, y			
≤50		1	
50‐60	**.002**	1.602	1.182‐2.173
60‐70	**.000**	2.273	1.679‐3.079
>70	**.000**	3.315	2.436‐4.510
Gender			
Male		1	
Female	.893	1.014	0.829‐1.240
Race, number (%)			
White		1	
Black	.468	0.842	0.529‐1.339
Others	.460	0.847	0.544‐1.317
Marital status			
Married (including common law)		1	
Divorced	.320	0.825	0.565‐1.205
Single (never married)	.073	1.318	0.975‐1.782
Separated	.828	1.121	0.399‐3.148
Widowed	.525	1.112	0.802‐1.542
Unknown	.323	0.729	0.389‐1.336
Grade			
Well differentiated		1	
Moderately differentiated	.317	3.307	0.317‐34.511
Poor differentiated	.986	1.019	0.127‐8.180
Undifferentiated	.962	0.952	0.125‐7.232
Unknown	.956	0.945	0.125‐7.153
Tumor size			
≤20 mm		1	
21‐40 mm	.285	1.319	0.794‐2.193
41‐60 mm	.171	1.416	0.861‐2.331
>60 mm	.072	1.668	0.956‐2.912
Unknown	**.011**	1.941	1.162‐3.240
Tumor extension			
Localized at one side, not cross the mid line		1	
Invasive, cross the mid line and distal metastasis	.393	1.137	0.847‐1.525
Unknown	.979	1.007	0.622‐1.628
Type of surgery			
None		1	
Partial resection	**.002**	0.462	0.282‐0.755
Partial resection NOS	**.033**	0.566	0.336‐0.954
Gross resection	**.011**	0.500	0.294‐0.852
Radical resection	**.006**	0.491	0.296‐0.813
Surgery NOS	**.045**	0.563	0.322‐0.987
Radiation after surgery			
Yes		1	
No	**.000**	1.908	1.495‐2.436

Bold indicates statistically significance.

Abbreviation: NOS, not otherwise specified.

**Table 5:** Final model

Variances	*P* value	Hazards ratio	95% confidence interval
Age, y	**.000**		
≤50		1	
50‐60	**.007**	1.357	1.087‐1.694
60‐70	**.000**	1.745	1.410‐2.160
>70	**.000**	2.898	2.321‐3.618
Type of surgery, number (%)			
None		1	
Partial resection	**.004**	0.592	0.413‐0.849
Partial resection NOS	**.012**	0.609	0.413‐0.899
Gross resection	**.014**	0.622	0.426‐0.907
Radical resection	**.005**	0.595	0.413‐0.856
Surgery NOS	.341	0.794	0.494‐1.276
Radiation after surgery			
Yes		1	
No	**.000**	1.961	1.655‐2.325
Tumor size	**.001**		
≤20 mm		1	
21‐40 mm	.111	1.367	0.931‐2.008
41‐60 mm	.058	1.445	0.988‐2.112
>60 mm	**.006**	1.813	1.188‐2.768
Unknown	**.001**	1.897	1.284‐2.801
Tumor extension	.107		
Localized at one side, not cross the mid line		1	
Invasive, cross the mid line and distal metastasis	**.040**	1.241	1.010‐1.525
Unknown	.726	0.933	0.634‐1.374

Bold indicates statistically significance.

Abbreviation: NOS, not otherwise specified.

### 3.3 Outcome and survival

**Figure 2:**
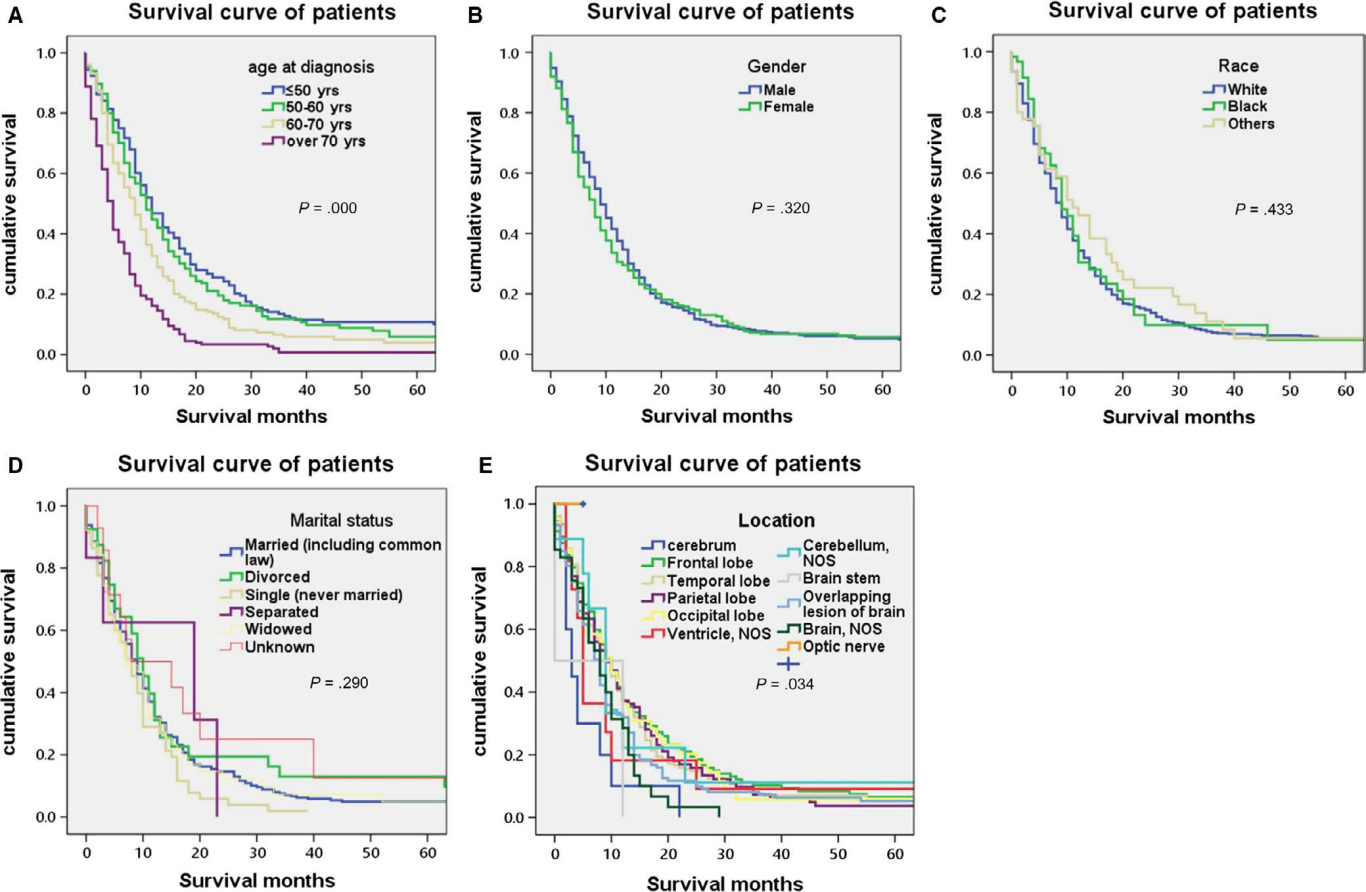
Kaplan‐Meier overall survival curves for single primary gliosarcoma patients by age at diagnosis (A); gender (B) and race (C); marital status (D). (E); Median survival for age ≤50 is 12 mo, 51‐60 is 11 mo; 61‐70 is 9 mo and >70 is 5 mo (*P* = .000 on log‐rank analysis). There is no significant difference in terms of gender (*P* = .320), race (*P* = .433) and marital status (*P* = .290). NOS, not otherwise specified

**Figure 3:**
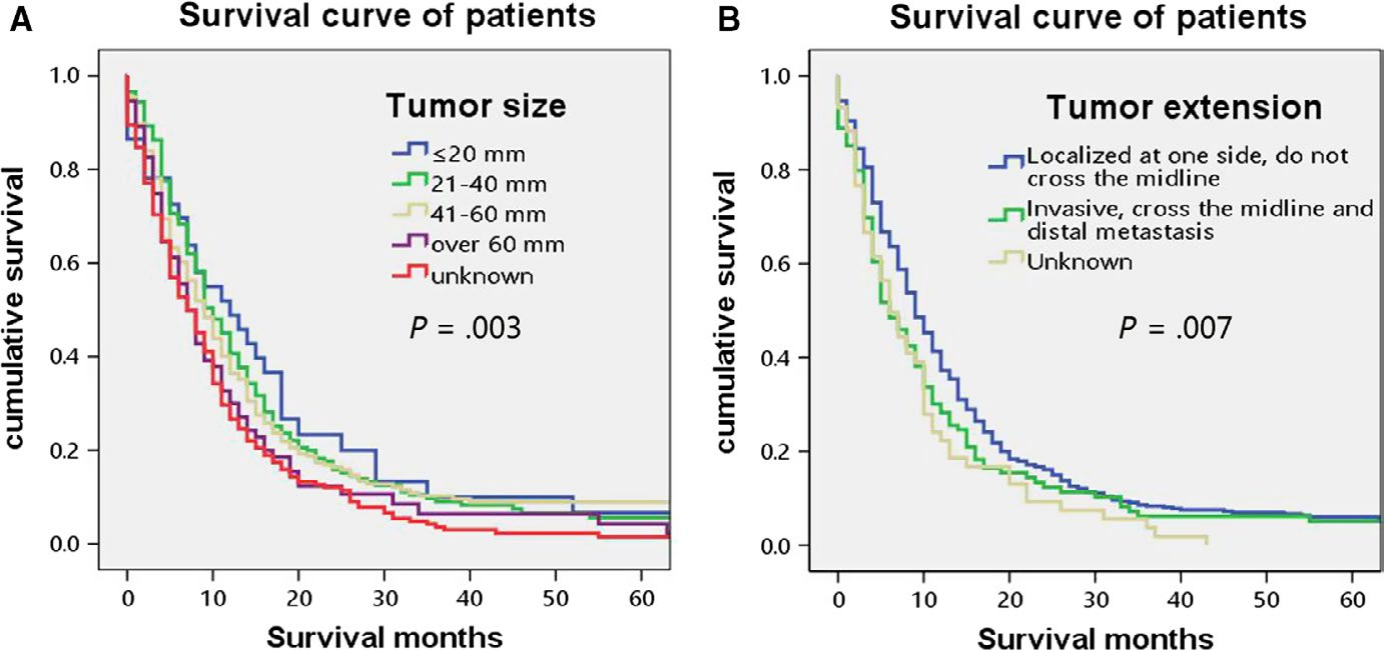
Kaplan‐Meier overall survival curves for single primary gliosarcoma patients by tumor size (A) and tumor extension (B). Median survival for tumor size ≤20 mm is 12 mo, 21‐40 mm is 10 mo; 41‐60 mm is 9 mo; >60 mm is 7 mo; and unknown group is 7 mo (*P* = .003 on log‐rank analysis). Median survival for tumor extension localized at one side and not cross the midline is 9 mo; invasive, cross the midline and distal metastasis is 6 mo and unknown is 6 mo (*P* = .007 on log‐rank analysis)

**Figure 4:**
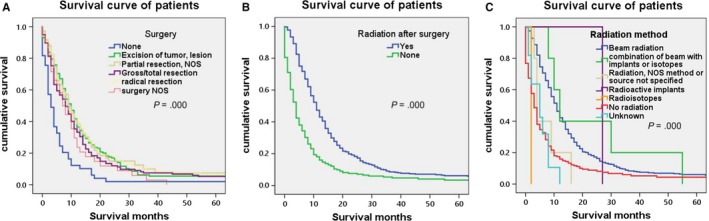
Kaplan‐Meier overall survival curves for GSM patients by type of surgery (A) and radiation after surgery (B). Radiation method (C) Median survival for none surgery is 3 mo, excision of tumor or lesion is 10 mo; partial resection not otherwise specified (NOS) is 9 mo, gross/total resection is 8 mo, radical resection is 9 mo and surgery NOS is 7 mo (*P* = .000 on log‐rank analysis). Median survival for radiation after surgery is 11 mo, none radiation after surgery is 4 mo (*P* = .000 on log‐rank analysis)

Overall survival rate was shown in Figure 1 with median survivals of 9 months in our research. Kaplan‐Meier OS curves for single PGS showed that the following variables, age at diagnosis, tumor size, tumor extension, type of surgery, and radiation after surgery, location, radiation method are most significantly associated with survival on univariate analysis, which are shown in Figures 2, 3, 4. The elder patients showed an obviously shortened survival in comparison to the younger group, with a 9 months (60‐70) and 5 months (>70) vs 12 months (≤50) and 11 months (50‐60), respectively (*P* = .000) (Figure 2A). No significant differences were observed in terms of gender, race, and marital status (Figure 2B‐D). Locations at frontal lobe (10 months), occipital lobe (10 months), temporal lobe (9 months), and parietal lobe (9 months) showed a better survival rate than other locations including cerebrum (3 months), ventricle NOS (5 months), or brain stem (0 months) (*P* = .034) (Figure 2E). Median survival for patient' tumor size ≤20 mm and 21‐40 mm was observed to be 12 and 10 months, which is better than that of size 41‐60 mm (9 months), >60 mm (7 months) and unknown group (7 months) (*P* = .003) (Figure 3A). Cases in tumor extension group 1 (localized at one side, not cross the midline) had a 9 months median survival, whereas patients in the tumor extension group 2 (invasive, cross the midline, and distal metastasis) had 6 months (*P* = .007 on log‐rank analysis) (Figure 3B). Surgery and radiation are critical treatment method for PGS. As is shown in Figure 4A, compared to none surgery group (3 months), patients who got surgery presented a prolonged survival (excision of tumor or lesion 10 months; partial resection 9 months; gross/total resection 8 months; radical resection 9 months) (*P* = .000). Furthermore, our research showed a fashion of survival benefit in patients undergoing radiation vs no radiation of 11 months vs 4 months, respectively (Figure 4B). Among all the radiation methods showed in Figure 4C, it is obvious that patients who received beam radiation (11 months) or a combination of beam with implants or isotopes (12 months) showed a longer survival time than other methods such as radiation NOS (4 months) or radioisotopes (2 months) (*P* = .000) (Figure 4C).

## 4 DISCUSSION

To data, there are only 13 articles that include over 10 patients of GSM.^4^, ^6^, ^8^, ^9^, ^10^, ^11^, ^15^, ^16^, ^17^, ^18^, ^19^, ^20^, ^21^ However, only one of them specified the diagnosis as PGS with a small cohort of patients.^17^ The rest of them did not distinguish primary and secondary GSM which could not represent the prognosis of single PGS. For example, Meis reported in 1991, among 26 GS patients and other GBM patients, that there were no significant differences between GS and GBM (8.3 and 9.6 months, respectively).^9^ Lutterbach reported that median survival of GS is 11.5 months and that of GBM is 8.1 months which did not reach statistical significance.^10^ Salvati et al reported the existence of two types of GS based on histology—predominantly gliomatous and predominantly sarcomatous. The latter one got a better survival rate.^16^ Seunggu J. Han proposed that patients with PGS resembling meningioma were found to have a significantly prolonged median survival compared with patients harboring PGS resembling GBM multiforme.^17^ Conversely, Kevin R. Kozak found that survival of GS was worse than GBM, which is contrary to Meis, Lutterbach.^10^ Only one article achieved a cohort over 300 samples but without distinguishing single PGS or secondary GS or GS in multiple sites.^18^ Besides, it excluded younger than 20 years' patients. According to that paper, the prognosis for GS patients appears slightly worse than that observed for GBM patients.

Primary gliosarcoma is a subtype of GBM and considered as a rare primary malignant central nervous system tumor.^19^ Primary gliosarcoma was identified as grade IV neoplasm in the 2007 World Health Organization classification.^22^ Primary gliosarcoma and GBM share similar symptoms.^8^ The most common clinical symptoms include headache and vomiting resulted from increased intracranial tension, accompanied by some aphasia, hemiparesis, seizures, and cognitive decline.^20^ They also share similar imaging characteristics such as peritumoral edema and mass effect.^4^ Several studies have found that temporal lobe was most frequent location for GS followed by frontal lobe.^19^ In our research, the most common sites of single PGS is cerebral including the temporal lobe, frontal lobe, overlapping lesion of brain, and parietal lobe. It can be concluded that single PGS, like GBM, has a tendency to localize at cerebral. However, rarely can single PGS be seen in the spinal cord.

Before we do our analysis, we did PH test to examine whether those prognostic factors are suitable for Cox proportional analysis model (data not shown). Results showed that PH test was positive (*P* > .05) indicating that our data are suitable for Cox proportional analysis model.

### 4.1 Age at diagnosis

Base on all the variables analyzed, we found that age at diagnosis, radiation after surgery, tumor size, and type of surgery were associated with survival of PGS patients. Robert A. Morantz reported in his clinical study of 24 cases that GS predominantly occurs between ages 40 and 70 years.^4^ Kevin R. Kozak found that GS has a tendency to influence the elderly patients.^10^ In our study, age at diagnosis of single PGS reached statistical significance in both univariate and multivariate analysis. Patients' age at diagnosis was divided into four groups, “≤50,” “51‐60,” “61‐70,” and “＞70” with median survival 12, 11, 9, and 5 months respectively. Our analysis revealed a strong correlation between age at diagnosis and survival of single PGS. Age at diagnosis could be considered as an independent influencing factor on survival of single PGS.

### 4.2 Tumor characteristics

Tumor size was shown to be statistically correlated with the survival of single PGS both in univariate and multivariate analysis. The larger the tumor was (>40 mm), the higher risk of survival the PGS would be. This result demonstrates that tumor size is positively associated with survival of PGS. However, other studies proposed that there were no significant differences in the size of PGS.^9^ As far as I am concerned, the discrepancy of these results was partly due to the sample size. The tumor extension reached significance just in univariate analysis indicating that it is not an independent influencing factor of single PGS.

### 4.3 Treatment

Knowledge about treatment of PGS was mainly arosed from case reports and small sample studies. At first, effective treatments used in GBM patients were applied in PGS.^6^ Surgery and radiation was supposed to be strongly associated with survival of PGS. Studies showed that GS patients who underwent no surgery (or biopsy only) achieved only 4 months which is shorter than those who underwent excision (median survival 7‐11 months).^10^ Some researchers found that median survival was longer in radical resection group than those in subtotal resection group.^18^ Conversely, other researchers also reported that there is no correlation between radical resection and prolonged survival in GBM.^21^ As a matter of fact, both the studies had their limitations due to their small cohort. Based on our large sample database, the patients who underwent no surgery had a poorer survival (3 months) than those who underwent surgeries. However, there is no significant difference in survival time between different types of surgical groups. The longest survival existed at excision of tumor or lesion group (10 months), followed by partial resection NOS (9 months), radical resection (9 months) and gross/total resection (8 months). It is reasonable to believe that surgical intervention is a predictor to prognosis of single PGS. Besides, it is crucial for patients to get surgery as their first treatment. Surgical intervention not only can be used to remove the tumor or lesion but also to identify the pathology of tumor to guide following treatment including chemotherapy and radiation.

Radiation is another predictor of single PGS survival. The effect of radiation is not clear in about 20 years ago. Robert A. Morantz, Perry and Parekh's small cohort studies showed that a majority of patients had undergone surgical resection while only a small portion of patients were given radiation therapy, and a few had received chemotherapy.^4^, ^20^, ^21^ In order to evaluate the benefit of radiation therapy on survival, Perry and his colleagues launched a cohort study.^21^ He found that radiation‐treated group had median survival of 10.6 months which is better than 6.25 months in patients not treated with radiation (P < .025). Nowadays, the application of radiation therapy for PGS learned from GBM because implementation of radiation has been well‐established in GBM.^10^, ^21^ The dose of radiation therapy for GBM varied from 45 to 81 Gy.^4^, ^9^, ^21^ It is insufficient to demonstrate the benefit of radiotherapy in PGS studied in small sample size researches. However, in our large cohort study, those who did not get radiation after surgery manifested a poorer prognosis. A median survival of 11 months was observed in radiation group compared to 4 months in none radiation group after surgery. Other studies also have mentioned different survival time in term of radiation given or not after surgery. Study by Kevin R. Kozak, also showed that postoperative radiation may improve OS rate of PGS patients. Besides, patients who got beam radiation or a combination of beam with implants or isotopes did showed a better survival rate. We have reason to believe that radiation is an independent predictor to single PGS.

## 5 LIMITATIONS

There are limitations in our study due to its inability to examine those factors not included into this database. For example, there is no information on occupation, gene mutation (isocitrate dehydrogenase) and chemotherapy, and its treatment frequency. Chemotherapy has become a standard method to treat glioma patients which has prolonged the life span. Data for the chemotherapy regimens and the cycles of chemotherapy, as well as radiation doses, were not provided in this database. Lacking of this part of data did impaired our knowledge of PGS. Lacking data about the subtype of PGS make us unable to evaluate survival of PGS resembling meningioma and PGS resembling GBM multiforme. The gene mutation was also not included in our study. For example, isocitrate dehydrogenase has been shown to play a key role in the classification and prognosis of glioma but do not exist in this database. Loss of those information has given a deficiency to our understanding of single PGS. Despite these limitations, further studies related to these factors are desperately needed.

## 6 CONCLUSION

Our research was the first large sample study of single PGS to fulfill our understanding about its demographics, treatment factors, and prognosis. This study indicated that single PGS has a poor prognosis. Outcome of single PGS would become poorer along with patients' age getting older and tumor size getting larger. Surgery intervention and radiation therapy were beneficial factors.

## CONFLICT OF INTEREST

The authors declare no conflict of interest.
